# *Veronicastrum wulingense* (Plantaginaceae), a new species from Southwestern Hubei, China

**DOI:** 10.1186/s40529-023-00367-y

**Published:** 2023-02-01

**Authors:** Shi-Xiong Ding, Hui Jiang, Jing Tian, Jing Ren, Fredrick Munyao Mutie, Emmanuel Nyongesa Waswa, Guang-Wan Hu, Qing-Feng Wang

**Affiliations:** 1 Core Botanical Gardens/Wuhan Botanical Garden, Chinese Academy of Sciences, Wuhan, 430074 China; 2 Jiangxi Provincial Key Laboratory for Bamboo Germplasm Resources and Utilization, Forestry College, Jiangxi Agricultural University, Nanchang, 330045 China; 3 Sino-Africa Joint Research Center, Chinese Academy of Sciences, Wuhan, 430074 China; 4 University of Chinese Academy of Sciences, Beijing, 100049 China; 5 Life Science College, Hunan Normal University, Changsha, 410081 China

**Keywords:** *Veronicastrum wulingense*, New species, Southwestern Hubei, Phylogeny, Wulingshan region

## Abstract

**Background:**

The genus *Veronicastrum* Heist. ex Fabr. are mainly distributed in East Asia, and only *Veronicastrum virginicum* (L.) Farw. is disjunctively distributed in eastern North America. The south area of China (extending to Taiwan Island) is the richest in *Veronicastrum* species. It is of medicinal importance in China as traditional herbs used to treat ascites diseases that caused by schistosomiasis. During field investigation of plant resources in Pingbaying National Forest Park, Southwestern Hubei, China, an unknown flowering population of *Veronicastrum* was discovered from thick humus layers adjacent to rocks under broad-leaved forests by walkways. They were collected and morphological characters assesed for further taxonomic treatment. Molecular analysis was also conducted to ascertain its phylogenetic position in the genus *Veronicastrum*.

**Results:**

This species is similar to *Veronicastrum liukiuense* (Ohwi) T.Yamaz. from the Ryukyu Islands, but can be distinctly differed by its axillary inflorescences (versus terminal on short leafy branches), pedicels up to 2.5 mm (versus sessile), corollas purple to purple-red (versus white tinged with pale purple) and florescence June to July (versus September to October). Also, phylogenetic studies showed the species was an independent clade in the genus *Veronicastrum* based on the maximum likelihood (ML) analyses using two different matrix sequences of concatenated molecular markers. The plastid genome of this new species is also reported in this study for the first time.

**Conclusion:**

The morphological and molecular evidences support the recognition of *Veronicastrum wulingense* as a new species.

**Supplementary Information:**

The online version contains supplementary material available at 10.1186/s40529-023-00367-y.

## Background

*Veronicastrum* Heist. ex Fabr. is a genus composed of perennial herbs in the family Plantaginaceae in Lamiales (The Angiosperm Phylogeny Group 2016; Li et al. 2021). It comprises about 20 species worldwide, which are mainly distributed in the East Asia (Hong et al. 1998), while only *Veronicastrum virginicum* (L.) Farw. is disjunctively distributed in the eastern North America. Morphologically, *Veronicastrum* species are closely similar to the genus *Veronica* L., but can be distinguished from *Veronica* by equally 5-lobed sepals, inside of corolla being densely hairy, reticulated ellipsoid seeds, hairy filaments which are usually longer than corolla (Yamazaki 1957; Albach and Chase 2001), and different pollen (Hong 1984). The genus is of medicinal importance in China as traditional herbs used to treat ascites diseases that caused by schistosomiasis (Chin and Hong 1979).

Previously, *Veronicastrum* species were placed in the genus *Veronica* in the tribe Veroniceae in Scrophulariaceae sensu lato, and later segregated from *Veronica* as a new genus by von Wettstein (1891) according to the significant morphological difference. However, these two genera, though separate, were still considered to be closely related (Albach and Chase 2001). The taxonomic position of *Veronicastrum* in Lamiales has changed considerably since its original circumscription with the wide application of molecular systematics. A series of molecular phylogenetic studies have revealed that the traditional delimitation of Scrophulariaceae was a complex polyphyly rather than monophyly, and found that Veroniceae was closely related to *Plantago* L. in almost all molecular analyses (Olmstead and Reeves 1995; Freeman and Scogin 1999; Olmstead et al. 2001; Albach et al. 2001; Xiao et al. 2020). Therefore, the Veroniceae clade was transferred to Plantaginaceae in the classifications of the Angiosperm Phylogeny Group II (APG II) (The Angiosperm Phylogeny Group 2003) and gradually accepted by the taxonomic domain (Albach et al. 2005; Tank et al. 2006).

In East Asia, the genus *Veronicastrum* is divided into four sections in *Flora Reipulblicae Popularis Sinicae*: Sect. Cdlorhabdos (Chin and Hong 1979), Sect. Plagiostachys, Sect. Pterocaulon, and Sect. Verouicastrum (Yamazaki 1957). In recent years, two new species of *V. loshanense* (Chen and Chou 2008) and *V. nogerchii* (Ueharai et al. 2013) were found and published from Taiwan Island (China) and Chiba Prefecture (Japan), respectively. Up to date, a total of 18 accepted *Veronicastrum* species are known worldwide according to The World Checklist of Vascular Plants (WCVP) (https://wcsp.science.kew.org/) (Govaerts et al. 2021).

When conducting field survey of plant resources in Pingbaying National Forest Park, Xianfeng County, Southwestern Hubei, China, in June, 2021, an interesting flowering population of *Veronicastrum* was found and collected on the thick humus layers adjacent to rocks under broad-leaved forests by walkways. The flowers were densely clustered at rachis apex and steadily ca. 2–3 cm long, and whole plants showed purple-red when young and covered by densely short curly hairs. Our identification was based on morphological characters using the available literature and herbarium specimens. After a detailed comparison with the known species, we ascertained that they represent a new taxon, which we hereby describe as *Veronicastrum wulingense.*

## Methods

### Morphological and taxonomic analyses

The morphological description of *Veronicastrum wulingense* was sourced from the observation and measurement of living plants, photographs taken during fieldwork, and designated type specimens. The whole living plants, inflorescences, and dissected flowers were carefully photographed. Comparison of morphological features was implemented between the new species and other related *Veronicastrum* species based on *Flora of China*, *Flora of Taiwan*, *Flora of Japan*, and other related literature. The specimen was examined from the virtual specimen from China and world’s major herbarium. Also, 12 *Veronicastrum* species materials from different places and *Pseudolysimachion spicatum* (L.) Opiz in China were collected, photographed, and specimens prepared (Additionl file 1: Table S1). The color photo plate and hand drawing illustration of the new species were sort and provided. The morphological comparison of diagnostic characteristics between *V. wulingense* and the similar species *V. liukiuense* (Ohwi) T.Yamaz. was carried out. The voucher specimens of the new species were deposited in the herbarium of Wuhan Botanical Garden (HIB), Chinese Academy of Sciences.

### Taxon sampling and DNA extraction, sequencing

In this study, 13 samples (10 taxa including *Veronicastrum wulingense* and *Pseudolysimachion spicatum* were newly sequenced to construct a phylogenetic tree to reveal the position of the new taxon in *Veronicastrum* (the voucher information of the 13 samples is listed in Additionl file 1: Table S1). Total genomic DNA was extracted from dry leaf materials that were kept in silica gels for preservation (Chase and Hills 1991) using a modified procedure of CTAB (cetyltrimethylammonium bromide) (Doyle and Doyle 1987; Li et al. 2013). The purified genomic DNA was fragmented to construct short-insert libraries for sequencing based on the Illumina paired-end technology platform (HiSeq-PE150 strategy) in the Novo gene Company (Beijing, China), and 6-GB reads of genome skimming data were obtained.

### Plastid genome assembly, annotation

Initially, the 16 plastid genomes were assembled using GetOrganelle v1.7.2 with appropriate parameters (Jin et al. 2020). The Bandage software was used to visualize the final assembly graphs to evaluate the completeness and accuracy of the assembled plastid genomes (Wick et al. 2015). Next, the assembled plastid genomes were annotated and two inverted repeat (IR) regions were found using PGA software with *Amborella trichopoda* (Accession: AJ506156.2) Baill. and *Veronicastrum axillare* (Accession: NC_056895.1) as the references (Qu et al. 2019). We then checked the annotated genes and protein-coding regions and corrected detected errors manually using Geneious-v10.2.3 (Kearse et al. 2012). The information of plastid genome features of *V. wulingense* was also analyzed in the software Geneious. The circular plastid genome map of *V. wulingense* was drawn and visualized in OGDRAW online software (https://chlorobox.mpimp-golm.mpg.de/OGDraw.html) (Accessed on 24 December 2021) (Greiner et al. 2019).

### Sequence alignment and phylogenetic analysis

The phylogenetic position of *Veronicastrum wulingense* in the genus *Veronicastrum* was analyzed based on the maximum likelihood (ML) method that was performed by the IQ-TREE program with 1000 bootstrap replications in the software PhyloSuite-v1.2.2 (Nguyen et al. 2015; Zhang et al. 2020; Minh et al. 2013). Two different phylogenetic trees were constructed to maximize the use of currently available and limited DNA data of *Veronicastrum* species from our collection and NCBI online database.

The aligned and concatenated molecular marker sequences of *matK*, *rbcL*, and *trnH-psbA* were selected to reconstruct phylogenetic tree in the tribe Veroniceae from 13 newly sequenced samples (Additional file 1: Table S1), 9 released plastid genomes (Additional file 1: Table S2) and four concatenated molecular marker sequences of plastid fragments (Additional file 1: Table S3) from the NCBI database using the program Concatenate Sequence in PhyloSuite-v1.2.2, of which outgroups were *Aragoa abietina* Kunth and *A. cleefii* Fern Alonso. The aligned marker sequences of *trnL-F* and ITS (internal transcribed spacer) were also concatenated to the other matrix dataset (Outgroup: *A. abietina*), including 13 newly sequenced samples and 17 concatenated molecular marker sequences from NCBI database (Additional file 1: Table S4). The software Partitionfinder 2 was used to find the best-fit partition model of the two different matrix sequences according to the Akaike information criterion (AIC) for ML analysis (Lanfear et al. 2017).

## Results and discussion

### Taxonomic treatment

#### *Veronicastrum wulingense* G.W. Hu & Q.F. Wang *sp. nov.* (Figs. 1, 2)

##### Type

CHINA. Hubei, Enshi Tujia and Miao Autonomous Prefecture, Xianfeng county, Pingbaying township, Pingbaying National Forest Park, Sidong Gorge, elev. 1324 m, 23 Jun, 2021, *S.X. Ding, H. Jiang*, *F.M. Mutie*, *G.W. Hu PBY-346* (holotype: HIB!, isotypes: PE, KUN, IBSC).

**Fig. 1:**
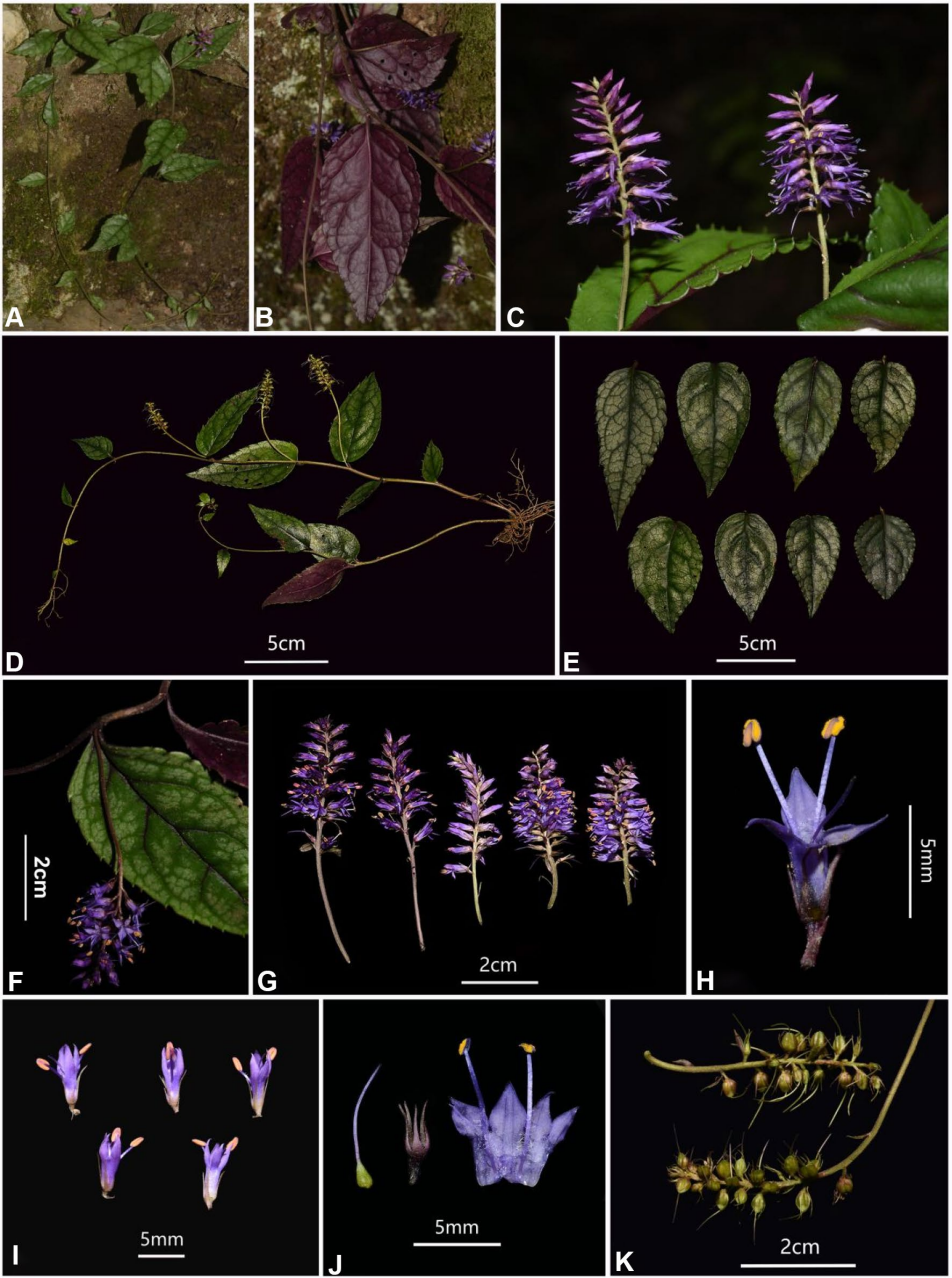
*Veronicastrum wulingense* (type locality). **A–B** habit; **C** leaf sawtooth; **D** individual; **E** leaves; **F **axillary inflorescence in the main axis;** G** inflorescences; **H–I** flowers; **J** anatomical flower; **K** infructescences. Photographed by S.X. Ding

**Fig. 2:**
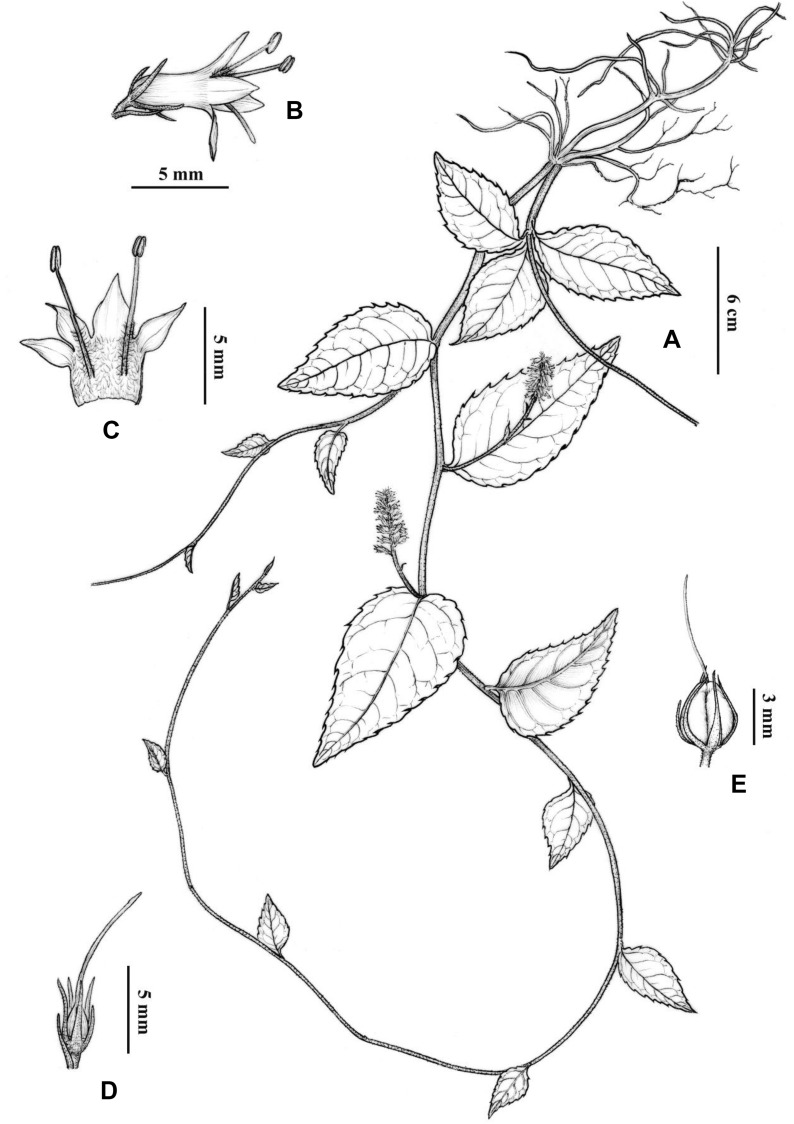
Illustration of *Veronicastrum wulingense*. **A** habit; **B** flower; **C** dissected corolla and stamens; **D** calyx and pistil; **E** capsule with persistent sepals. Drawn by J. Tian

##### Diagnosis

*Veronicastrum wulingense* belongs to the typical *Veronicastrum* species of axillary inflorescences (Sect. Plagiostachys species). Morphologically, it is closely similar to *V. liukiuense* from the Ryukyu Islands, but can be distinctly differed by its axillary inflorescences (versus terminal on short leafy branches), pedicels up to 2.5 mm (versus sessile), corollas purple to purple-red (versus white tinged with pale purple), and florescence June to July (versus September to October) (Table 1). Also, these diagnostic characteristics of peduncles up to 7 cm, flowers clustered densely in rachis apex and steadily ca. 2–3 cm long, which are unique in the all known *Veronicastrum* species of axillary inflorescences.

**Table 1:** Morphological comparison of the diagnostic characteristics between *Veronicastrum wulingense* and *V. liukiuense*

Characters	*Veronicastrum wulingense*	*Veronicastrum liukiuense*
Plant habit	Arching and rooting apically	Ascending below, arching and rooting apically
Stem	Terete, not angular, with densely short curly hairs	Striate, sparsely pubescent
Petiole	Densely short curly hairs	Pubescent
Leaf	Ovate to ovate-lanceolate, 3–12 × 2–6 cm, blade abaxially purple-red, sparse white pubescent to nearly glabrous	Blade ovate or orbiculate ovate, 5–9 × 2.5–7 cm, lower surface sparsely hirsute on nerves
Serration	Undate, irregular, cuspidate to long apiculate dentate, apex upward, or crenate dentate	Depressed mucronate teeth
Inflorescence	Axillary, 4–9 cm, peduncle 1–7 cm, rachis densely short curly hairs, flower cluster dense in peduncle apex, steadily ca. 2–3 cm	Terminal on short leafy branches, 2–4 cm, 1.5 cm cross in flowers, peduncle hirsute
Bracts and calyx lobes	Bracts and calyx lobes linear-lanceolate to narrowly triangular, conspicuously shorter than corolla, densely short ciliate	Bracts linear-lanceolate, 6–8 mm, acuminate, hirsute; calyx lobes lanceolate, attenuate acuminate, ca. 4 mm, sparsely pilose on upper margin
Flower	Purple-red, pedicellate to subsessile (0–2.5 mm) with densely short curly hairs, corolla 5.5–7 mm	White tinged with pale purple, sessile, corolla ca. 7 mm
Corolla lobes	Ovate-triangular to narrowly triangular, 2/5–1/2 of corolla length	Triangular-lanceolate, acute, ca. 3 mm
Florescence	June to July	September to October

##### Description

Herbs, perennial. Rhizomes short, horizontal. Stems densely short curly hairs, up to 90 cm long, terete, without angles and strias, basally branched, arching and rooting apically. Leaves alternate, petioles short, often purple-brown, no more than 5 mm long, with short curly hairs. Leaf blade thick papery to leathery, ovate to ovate-lanceolate, 3–12 × 2–6 cm; base rounded, rarely broadly cuneate, apex acute to shortly acuminate; margin serrations undate, irregular, cuspidate to long apiculate dentate, apex upward, or crenate. Leaf blades abaxially purple-red, sparse white pubescent to nearly glabrous on both surfaces, densely on veins; veins clear, lateral veins 4 − 6 pairs, concave on adaxial surface and convex on abaxial surface. Spicate inflorescences axillary, 4–9 cm long; peduncles 1–7 cm long, usually surrounded by several small leafy involucrate bracts in middle-upper part; flowers pedicellate to subsessile (0–2.5 mm long**)**, densely clustered at rachis apex, steadily ca. 2–3 cm long; peduncles, rachis, and pedicels densely short curly hairs. Calyxes deeply 5-lobed, one adaxial lobe smaller, bracts and calyx lobes linear-lanceolate to narrowly triangular, conspicuously shorter than corolla, densely short ciliate. Corollas purple to purple-red, 5.5–7 mm long; corolla tubes tubular, straight, inner surface crinite; corollas equally 4-lobed, lobes all straight, actinomorphic, ovate-triangular to lanceolate, 2/5–1/2 of corolla length. Stamens 2, slightly to conspicuously exserted, exceeding corolla by ca. 2–3 mm long, crinite at lower middle part; anthers orange-yellow, oblong, 1–1.5 mm long; anther locules connivent, not confluent; ovaries glabrous, stigmas small and slightly dilated, styles 5–7 mm long. Capsules ovoid-globose, 3–3.5 mm long, 2-grooved, 4-valved; style, bracts, and calyx persistent. Seeds numerous per capsule, small, oblong, seed coat reticulate. Fl. Jun–Jul, Fr. Aug–Oct.

##### Distribution and habitat

The new species *Veronicastrum wulingense* is currently known only from Sidong Gorge of Pingbaying National Forest Park in the Northcentral of Wulingshan Region, Southwestern Hubei, China—its type locality. All our collections were made from the thick humus layers adjacent to rocks under broad-leaved forests by walkways, at elevations of 1000–1400 m above sea level.

##### Etymology

The specific epithet ‘*wulingense*’ refers to Wulingshan Region, where the new species is distributed. The Chinese name is ‘Wu Ling Fu Shui Cao (武陵腹水草)’.

##### Phenology

Based on our field surveys, the new species was observed flowering from June to July, and fruiting from August to October.

##### Conservation Significance

 *Veronicastrum wulingense* is an endemic species and currently only known from the type locality in China. It has narrow distribution area is very narrow and only known few from populations. Therefore, we recommended that this species be treated as a protected plant in China, as well as the protection of its habitat.

### The phylogenetic position of *Veronicastrum wulingense*

**Fig. 3:**
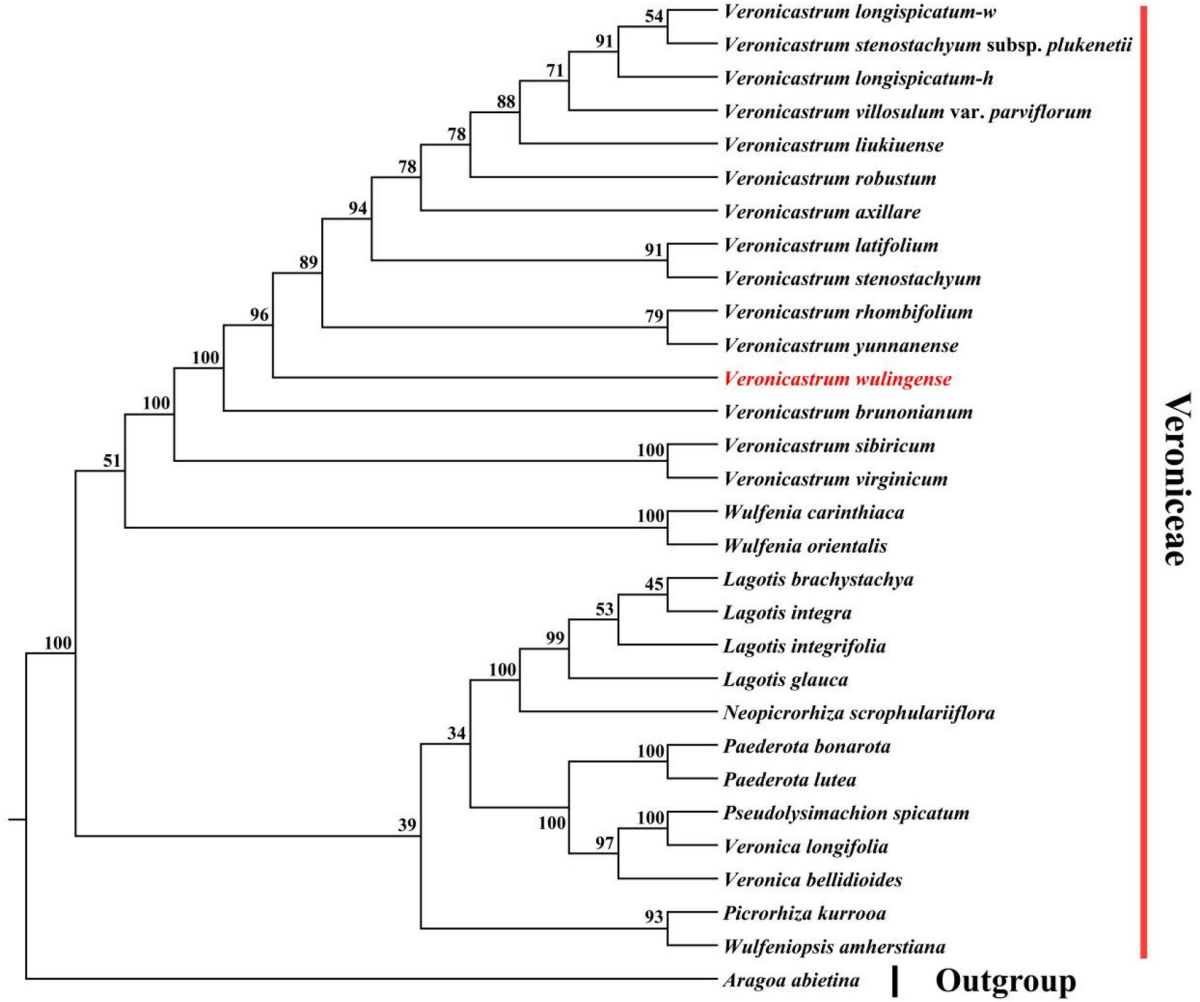
The phylogenetic position of *V. wulingense* in the genus *Veronicastrum* based on the maximum likelihood (ML) method using 30 concatenated matrix sequences from ITS (internal transcribed spacer) and plastid fragment *trnL-F*.

**Fig. 4:**
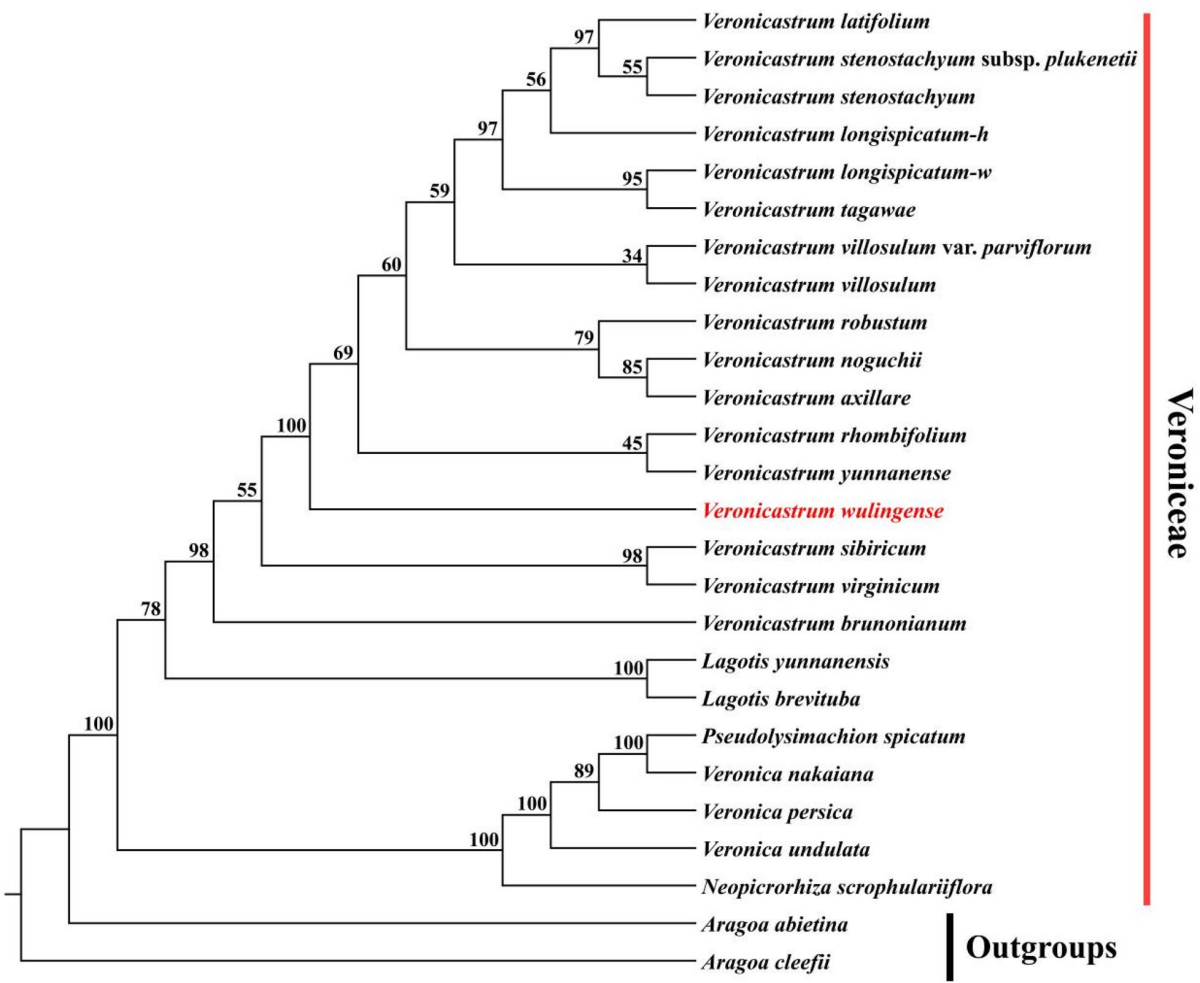
The phylogenetic position of *V. wulingense* in the genus *Veronicastrum* based on ML method using 26 concatenated matrix sequences from three plastid molecular markers

The phylogenetic position of *Veronicastrum wulingense* was revealed in the genus *Veronicastrum* with high support values in the ML trees based on two different matrix sequences of concatenated molecular markers (Figs. 3, 4). The results displayed that *V. wulingense* is an independent clade and related to *V. rhombifolium* (Hand.-Mazz.) Tsoong and *V. yunnanense* (W. W. Smith) Yamazaki in the two phylogenetic trees, while distant from the morphologically similar species *V. liukiuense* (Fig. 3). The molecular evidence support the recognition of *V. wulingense* as a new species. Besides, the species of *V. longispicatum* (Merr.) Yamazaki, *V. latifolium* (Hemsl.) Yamazaki, and *V. stenostachyum* (Hemsl.) Yamazaki (*V. stenostachyum* subsp. *plukenetii*), which are morphologically similar, showed unclearly and crossed interspecific relationships. We also found that the clade of *Pseudolysimachion spicatum* is nested in the clades of genus *Veronica* and verified *Veronica* is a paraphyly genus (Chen et al. 2020).

### The plastid genome features of *Veronicastrum wulingense*

**Fig. 5:**
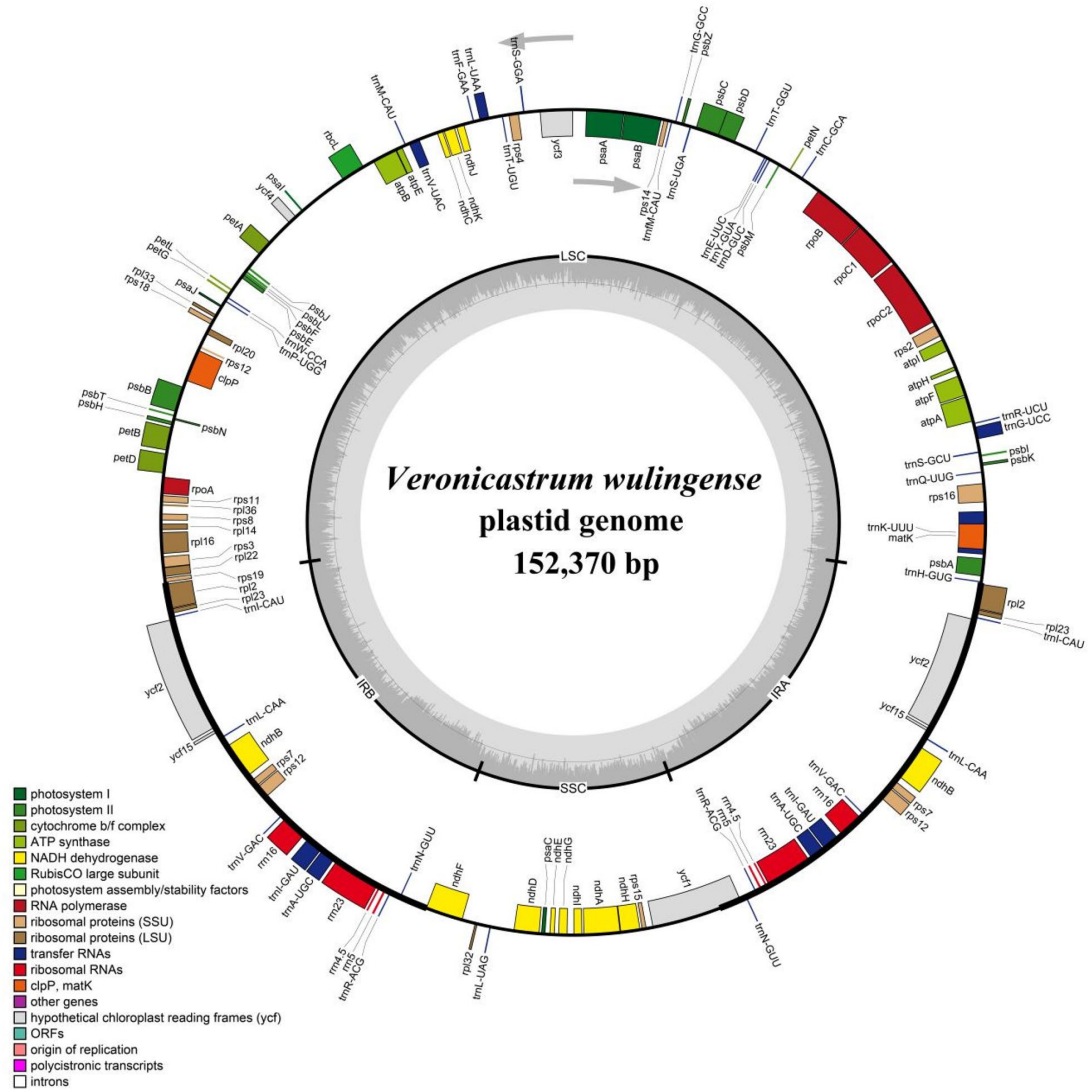
Circular plastid genome map of *Veronicastrum wulingense*. Genes drawn within the circle are transcribed clockwise, while those drawn outside are transcribed counterclockwise. Genes of different functional groups are colored by different colors. The darker gray in the inner circle corresponds to the DNA GC content, while the lighter gray corresponds to the DNA AT content

The complete plastid genome of *Veronicastrum wulingense* is 152,370 bp in length and portrays a circular and quadripartite structure, typical of most angiosperms (Fig. 5). The genome encompass a large single-copy (LSC) region (87,034 bp) and a small single-copy (SSC) region (18,492 bp), which are separated by a pair of inverted repeats (IRs) regions (25,980 bp). The GC content is 38.3% in the whole plastid genome, while that in the LSC, SSC, and IR regions are 36.5%, 32.3%, and 43.3%, respectively, which is relatively higher than the reported *Veronica persica* Poir. and *V. nakaiana* Ohwi. (a closely related genus of *Veronicastrum*) in LSC and SSC regions (Choi et al. 2016). The GC content in IR regions is significantly higher than that in LSC and SSC, which may be due to the tRNA and rRNA genes that occupy a higher proportion of the regions and have a relatively higher GC content. A total of 132 functional genes were annotated in the plastid genome of *V. wulingense* and can be divided into four categories and subdivided into 18 groups (Table 2), which include 87 protein-coding genes (PCGs), 37 tRNA genes, and 8 rRNA genes (duplicated in two IR regions: 7 PCGs, 7 tRNA genes, and 4 rRNA genes). Also, 18 genes with introns were annotated, including 12 PCGs (*clpP* and *ycf3* genes contain two introns) and six tRNA genes. Additionally, the start codon of *rps19* gene transcription mutated and is non-canonical “GTG” instead of the most common “ATG”.

**Table 2:** List of the annotated genes in the chloroplast genomes of *Veronicastrum wulingense*

Category	Groups of genes	Name of genes
Self-replication	Ribosomal RNA	*rrn4.5* ^c^, *rrn5* ^c^, *rrn16* ^c^, *rrn23* ^c^
	Transfer RNA	*trnA-UGC* ^a,c^, *trnC-GCA*, *trnD-GUC*, *trnE-UUC*, *trnF-GAA*, *trnG-GCC*, *trnG-UCC* ^a^, *trnH-GUG*, *trnI-CAU* ^c^, *trnI-GAU* ^*a,c*^, *trnK-UUU* ^a^, *trnL-CAA* ^c^, *trnL-UAA* ^a^, *trnL-UAG*, *trnM-CAU*, *trnfM-CAU*, *trnN-GUU* ^c^, *trnP-UGG*, *trnQ-UUG*, *trnR-UCU*, *trnR-ACG* ^c^, *trnS-UGA*, *trnS-GCU*, *trnS-GGA*, *trnT-GGU*, *trnT-UGU*, *trnV-UAC* ^a^, *trnV-GAC* ^c^, *trnW-CCA*, *trnY-GUA*
	Small subunit of ribosome	*rps2*, *rps3*, *rps4*, *rps7* ^c^, *rps8*, *rps11*, *rps12* ^a,c^, *rps14*, *rps15*, *rps16* ^a^, *rps18*, *rps19*
	Large subunit of ribosome	*rpl2* ^a,c^, *rpl14*, *rpl16* ^a^, *rpl20*, *rpl22*, *rpl23* ^c^, *rpl32*, *rpl33*, *rpl36*
	RNA polymerase subunits	*rpoA*, *rpoB*, *rpoC1* ^a^, *rpoC2*
Photosynthesis	Photosystem I	*psaA*, *psaB*, *psaC*, *psaI*, *psaJ*
	Photosystem II	*psbA*, *psbB*, *psbC*, *psbD*, *psbE, psbF*, *psbH*, *psbI*, *psbJ*, *psbK*, *psbL*, *psbM*, *psbN*, *psbT*, *psbZ*
	Subunits of cytochrome	*petA*, *petB* ^a^, *petD* ^a^, *petG*, *petL*, *petN*
	ATP synthase	*atpA*, *atpB*, *atpE*, *atpF* ^a^, *atpH*, *atpI*
	NADH-dehydrogenase	*ndhA* ^a^, *ndhB* ^a,c^, *ndhC*, *ndhD*, *ndhE*, *ndhF*, *ndhG*, *ndhH*, *ndhI*, *ndhJ*, *ndhK*
Other genes	Rubisco large subunit	*rbcL*
	Translational initiation factor	*infA*
	Maturase K	*matK*
	Envelope membrane protein	*cemA*
	Acetyl-CoA carboxylase	*accD*
	Proteolysis	*clpP* ^b^
	Cytochrome c biogenesis	*ccsA*
Unknown	Conserved open reading frames	*ycf1*, *ycf2* ^c^, *ycf3* ^b^, *ycf4*, *ycf15* ^c^

^a^Genes with one intron

^b^Genes with two introns

^c^ Two gene copied in IR regions

## Supplementary information

**Additional file 1: Table S1. **The vouchers of 13 samples of genome skimming. **Table S2.** 9 plastid genomesfrom NCBI database. **Table S3. **4concatenated sequences based on three DNA plastome fragments from NCBI database. **Table S4.** 17 concatenatedsequences based on two DNA fragments from NCBI database.

## Data Availability

Not applicable.

## Abbreviations

HIB: Wuhan Botanical Garden, Chinese Academy of Sciences, Herbarium
PE: Institute of Botany, the Chinese Academy of Sciences, Herbarium
KUN: Kunming Institute of Botany, Chinese Academy of Sciences, Herbarium
IBSC: South China Botanical Garden, Chinese Academy of Sciences, Herbarium
ML: Maximum likelihood
LSC: Large single-copy
SSC: Small single-copy
IR: Inverted repeat
PCG: Protein-coding gene

## References

[CR1] Albach DC, Chase MW (2001). Paraphyly of *Veronica* (Veroniceae; Scrophulariaceae): evidence from the Internal transcribed spacer (ITS) sequences of nuclear ribosomal DNA. J Plant Res.

[CR2] Albach DC, Soltis PS, Soltis DE, Olmstead RG (2001). Phylogenetic analysis of asterids based on sequences of four genes. Ann Mo Bot Gard.

[CR3] Albach DC, Meudt HM, Oxelman B (2005). Piecing together the “new” Plantaginaceae. Am J Bot.

[CR4] Chase MW, Hills HH (1991). Silica gel: an ideal material for field preservation of leaf samples for DNA studies. Taxon.

[CR5] Chen TT, Chou FS (2008). A new Taiwan species *Veronicastrum loshanense *(Scrophulariaceae). Bot Stud.

[CR6] Chen ZD, Lu AM, Liu B, Ye JF (2020). Tree of life for chinese vascular plants.

[CR7] Chin TL, Hong DY, Tsong PC, Yang HP (1979). Scrophulariaceae. Flora Reipublicae Popularis Sinicae.

[CR8] Choi KS, Chung MG, Park S (2016). The complete chloroplast genome sequences of three veroniceae species (Plantaginaceae): comparative analysis and highly divergent regions. Front Plant Sci.

[CR9] Doyle JJ, Doyle JL (1987). A rapid DNA isolation procedure for small quantities of fresh leaf tissue. Phytochem Bull.

[CR10] Freeman CE, Scogin R (1999). Potential utility of chloroplast trnL (UAA) gene intron sequences for inferring phylogeny in Scrophulariaceae. Aliso.

[CR11] Govaerts R, Lughadha EN, Black N, Turner R, Paton A (2021). The World checklist of vascular plants, a continuously updated resource for exploring global plant diversity. Sci Data.

[CR12] Greiner S, Lehwark P, Bock R (2019). OrganellarGenomeDRAW (OGDRAW) version 1.3.1: expanded toolkit for the graphical visualization of organellar genomes. Nucleic Acids Res.

[CR13] Hong DY (1984). Taxonomy and evolution of the Veroniceae (Scrophulariaceae) with special reference to palynology.

[CR14] Hong DY, Yang HB, Jin CL, Jin CL, Noel HH, Wu ZY, Raven PH (1998). Scrophulariaceae. Flora of China.

[CR15] Jin JJ, Yu WB, Yang JB, Song Y, dePamphilis CW, Yi TS, Li DZ (2020). GetOrganelle: a fast and versatile toolkit for accurate de novo assembly of organelle genomes. Genome Biol.

[CR16] Katoh K, Standley DM (2013). MAFFT multiple sequence alignment software version 7: improvements in performance and usability. Mol Biol Evol.

[CR17] Kearse M, Moir R, Wilson A, Stones-Havas S, Cheung M, Sturrock S, Buxton S, Cooper A, Markowitz S, Duran C (2012). Geneious basic: an integrated and extendable desktop software platform for the organization and analysis of sequence data. Bioinformatics.

[CR18] Lanfear R, Frandsen PB, Wright AM, Senfeld T, Calcott B (2017). PartitionFinder 2: new methods for selecting partitioned models of evolution for molecular and morphological phylogenetic analyses. Mol Biol Evol.

[CR19] Li J, Wang S, Yu J, Wang L, Zhou S (2013). A modified CTAB protocol for plant DNA extraction. Chin Bull Bot.

[CR20] Li HT, Luo Y, Gan L, Ma PF, Gao LM, Yang JB, Cai J, Gitzendanner MA, Fritsch PW, Zhang T (2021). Plastid phylogenomic insights into relationships of all flowering plant families. BMC Biol.

[CR21] Minh BQ, Nguyen MA, von Haeseler A (2013). Ultrafast approximation for phylogenetic bootstrap. Mol Biol Evol.

[CR22] Nguyen LT, Schmidt HA, von Haeseler A, Minh BQ (2015). IQ-TREE: a fast and effective stochastic algorithm for estimating maximum-likelihood phylogenies. Mol Biol Evol.

[CR23] Olmstead RG, Reeves PA (1995). Evidence for the Polyphyly of the Scrophulariaceae based on chloroplast *rbcL* and *ndhF* sequences. Ann Mo Bot Gard.

[CR24] Olmstead RG, Pamphilis CW, Wolfe AD, Young ND, Elisons WJ, Reeves PA (2001). Disintegration of the Scrophulariaceae. Am J Bot.

[CR25] Qu XJ, Moore MJ, Li DZ, Yi TS (2019). PGA: a software package for rapid, accurate, and flexible batch annotation of plastomes. Plant Methods.

[CR26] Tank DC, Beardsley PM, Kelchner SA, Olmstead RG (2006). Review of the systematics of Scrophulariaceae s.l. and their current disposition. Aust Syst Bot.

[CR27] The Angiosperm Phylogeny Group (2003). An update of the Angiosperm Phylogeny Group classification for the orders and families of flowering plants APG II. Bot J Linn Soc.

[CR28] The Angiosperm Phylogeny Group (2016). An update of the Angiosperm Phylogeny Group classification for the orders and families of flowering plants: APG IV. Bot J Linn Soc.

[CR29] Uehara K, Saiki K, Ando T (2013). *Veronicastrum noguchii* (sect. Plagiostachys, Plantaginaceae), a new species from Japan. Acta Phytotax Geobot.

[CR30] von Wettstein R, Engler A, Prantl K (1891). Scrophulariaceae. Die naturlichen Pflanzenfamilien, IV, 3b.

[CR31] Wick RR, Schultz MB, Zobel J, Holt KE (2015). Bandage: interactive visualization of de novo genome assemblies. Bioinformatics.

[CR32] Xiao J, Wang X, Li C, Liu H, Chen F (2020). Phylogeny of *Rehmannia* and related genera based on chloroplast genome. Mol Plant Breed.

[CR33] Yamazaki T (1957). Taxonomical and phylogenetic studies of Scrophulariaceae-Veronicae with special reference to *Veronica* and *Veronicastrum* in eastern Asia. J Fac Sci Univ Tokyo Sect 3 Bot.

[CR34] Zhang D, Gao F, Jakovlić I, Zou H, Zhang J, Li WX, Wang GT (2020). PhyloSuite: an integrated and scalable desktop platform for streamlined molecular sequence data management and evolutionary phylogenetics studies. Mol Ecol Resour.

