# Supplementary material for: Veronicastrum wulingense (Plantaginaceae), a new species from Southwestern Hubei, China
Source: Bot Stud. 2023 Feb 1;64:3. doi: 10.1186/s40529-023-00367-y (PMC9889593; doi:10.1186/s40529-023-00367-y)
Supplement: Supplementary file 1 — Additional file 1: Table S1. The vouchers of 13 samples of genome skimming. Table S2. 9 plastid genomesfrom NCBI database. Table S3. 4concatenated sequences based on three DNA plastome fragments from NCBI database. Table S4. 17 concatenatedsequences based on two DNA fragments from NCBI database. [file 40529_2023_367_MOESM1_ESM.docx]

**Table S1.** The vouchers of 13 samples of genome skimming.

| Species Name | Location in China | Collection Number | Herbarium |
| --- | --- | --- | --- |
| *Pseudolysimachion spicatum* | Haidian, Beijing | wbgd-164 | HIB |
| *Veronicastrum brunonianum* | Pengzhou, Sichuan | wbgd-86 | HIB |
| *Veronicastrum latifolium* | Wenchuan, Sichuan | HGW-001488 | HIB |
| *Veronicastrum longispicatum-h* | Yongzhou, Hunan | wbgd-170 | HIB |
| *Veronicastrum longispicatum-w* | Wuhan, Hubei | wbgd-1 | HIB |
| *Veronicastrum rhombifolium* | Baoxing, Sichuan | wbgd-112 | HIB |
| *Veronicastrum robustum* | Zixi, Jiangxi | wbgd-148 | HIB |
| *Veronicastrum sibiricum* | Mentougou, Beijing | CPG-67189 | PE |
| *Veronicastrum stenostachyum* subsp. *plukenetii* | Xinxian, Henan | DC-97 | HIB |
| *Veronicastrum stenostachyum* | Guzhang, Hunan | wbgd-147 | HIB |
| *Veronicastrum villosulum* var. *parviflorum* | Nanjing, Jiangsu | wbgd-165 | HIB |
| *Veronicastrum yunnanense* | Wuding, Yunnan | wbgd-142 | HIB |
| *Veronicastrum wulingense* | Xianfeng, Hubei | PBY-346 | HIB |

**Table S2.** 9 plastid genomes from NCBI database.

| Species Name | Accession |
| --- | --- |
| *Aragoa abietina* | MW877561 |
| *Aragoa cleefii* | MW877562 |
| *Lagotis brevituba* | MW182582 |
| *Lagotis yunnanensis* | MN752238 |
| *Neopicrorhiza scrophulariiflora* | NC_057075 |
| *Veronica nakaiana* | NC_031153 |
| *Veronica persica* | NC_031344 |
| *Veronica undulata* | MW783683 |
| *Veronicastrum axillare* | MW244757 |

**Table S3.** 4 concatenated sequences based on three DNA plastome fragments from NCBI database.

| Name | Accession | | |
| --- | --- | --- | --- |
|  | *psbA-trnH* | *matK* | *rbcL* |
| *Veronicastrum noguchii* | LC586364 | LC586356 | LC586342 |
| *Veronicastrum tagawae* | LC586368 | LC586358 | LC586347 |
| *Veronicastrum villosulum* | LC586368 | LC586357 | LC586345 |
| *Veronicastrum virginicum* | FJ848087 | MK520801 | KX037379 |

**Table S4.** 17 concatenated sequences based on two DNA fragments from NCBI database.

| Name | Accession | |
| --- | --- | --- |
|  | ITS | *trnL-F* |
| *Aragoa abietina* | AJ459404.1 | MW877561 |
| *Lagotis brachystachya* | AF313027.1 | KC413573.1 |
| *Lagotis glauca* | KC237785.1 | KC413583.1 |
| *Lagotis integrifolia* | KJ630574.1 | KC413595.1 |
| *Lagotis integra* | KC237786.1 | KC413588.1 |
| *Neopicrorhiza scrophulariiflora* | EU078906.1 | KC413606.1 |
| *Picrorhiza kurrooa* | AF509813.1 | AF486414.1 |
| *Paederota lutea* | AF313024.1 | AF486408.1 |
| *Paederota bonarota* | AF509812.1 | AF513353.1 |
| *Wulfeniopsis amherstiana* | FJ848064.1 | AF486411.1 |
| *Wulfenia carinthiaca* | AF313025.1 | AF486409.1 |
| *Wulfenia orientalis* | KC237783.1 | AF486410.1 |
| *Veronica longifolia* | KJ630608.1 | KJ630705.1 |
| *Veronica bellidioides* | AF313010.1 | DQ232750.1 |
| *Veronicastrum axillare* | EU224210.1 | EU233624.1 |
| *Veronicastrum virginicum* | AF313030.1 | KX061021.1 |
| *Veronicastrum liukiuense* | AF509815.1 | AF486413.1 |
